# Direct observation of spin-resolved valence band electronic states from a buried magnetic layer with hard X-ray photoemission

**DOI:** 10.1080/14686996.2021.1912576

**Published:** 2021-05-13

**Authors:** Shigenori Ueda, Yuya Sakuraba

**Affiliations:** a Research Center for Functional Materials, National Institute for Materials Science (NIMS), Tsukuba, Japan; b Research Center for Advanced Measurement and Characterization, NIMS, Tsukuba, Japan; c Synchrotron X-ray Station at SPring-8, NIMS, Hyogo, Japan; d Research Center for Magnetic and Spintronic Materials, NIMS, Tsukuba, Japan

**Keywords:** Hard X-ray photoelectron spectroscopy (HAXPES), bulk-sensitive electronic state, spin-resolved valence band photoemission, buried ferromagnetic material, 40 opticalmagnetic electronic device materials, 203 magnetics/spintronics/superconductors, 502 electron spectroscopy, 700 others (development of spin-resolved HAXPES)

## Abstract

We report spin-resolved hard X-ray photoelectron spectroscopy (spin-HAXPES) for a buried Fe thin film in the valence band region. For the spin-HAXPES experiments, we developed an ultracompact built-in Mott-type spin-filter in a sample carrier, which enabled us to use the merit of two-dimensional (2D) multi-channel detector in a recent photoelectron analyser without modifying an apparatus for HAXPES. The effective Sherman function and the single-channel figure of merit (FOM) of the spin-filter were assessed to be −0.07 and 2.0 × 10^−4^, respectively. By utilizing the 2D detector of the photoelectron analyser, the effective FOM increased by a factor of ~4 × 10^4^ compared to the case when only 1 channel of the 2D detector was used. We have applied spin-HAXPES to MgO(2 nm)/Fe(50 nm)/MgO(001) structures. The spin-HAXPES experiments revealed the majority and minority spin electronic states and the spin polarisation of the buried Fe thin film. Due to the large photoionization cross-section of the 4*s* orbital of Fe in HAXPES, the spin-resolved spectra mainly reflected the Fe 3*d* and 4*s* states. The observed spin-HAXPES and spin polarisation spectral shapes agreed well with the calculated spin-resolved cross-section weighted densities of states and spin polarisation spectra. In contrast, a small discrepancy in the energy scale was recognised due to the electron correlation effects. These results suggest that the electron correlation effects are important in the electronic structure of bulk Fe, and spin-HAXPES is useful for detecting genuine spin-resolved valence band electronic structures of buried magnetic materials.

## Graphical abstract

**Figure:**
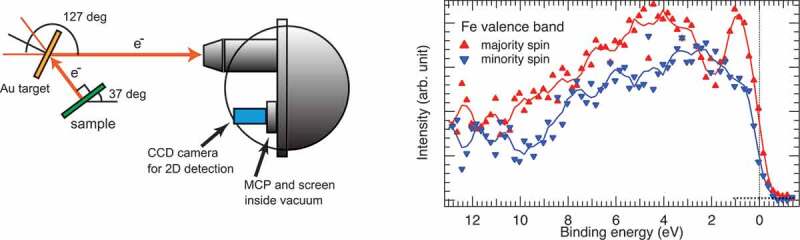

## 1. Introduction

**Figure 1.**
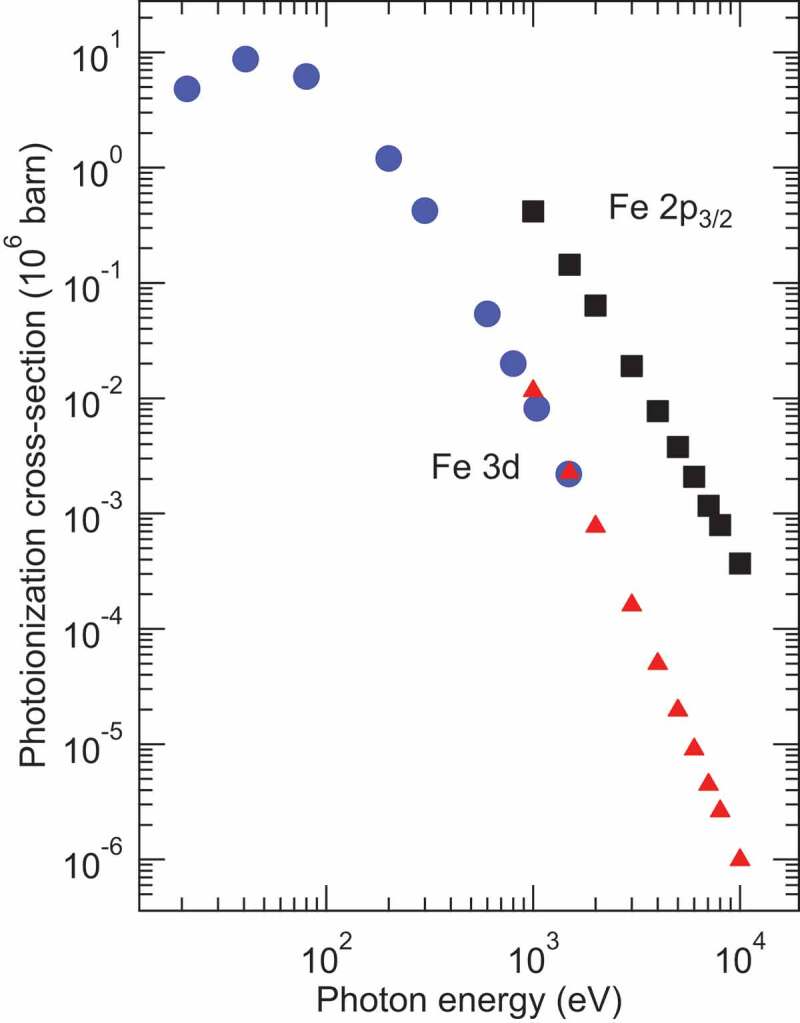
Theoretical photoionization cross-section in the energy range between 20 and 10^4^ eV for the isolated Fe atom. The cross-section of the Fe 3*d* orbital is indicated by circles [5] and triangles [6]. The cross-section of the Fe 2*p*_3/2_ orbital is indicated by squares [6]

**Figure 2.**
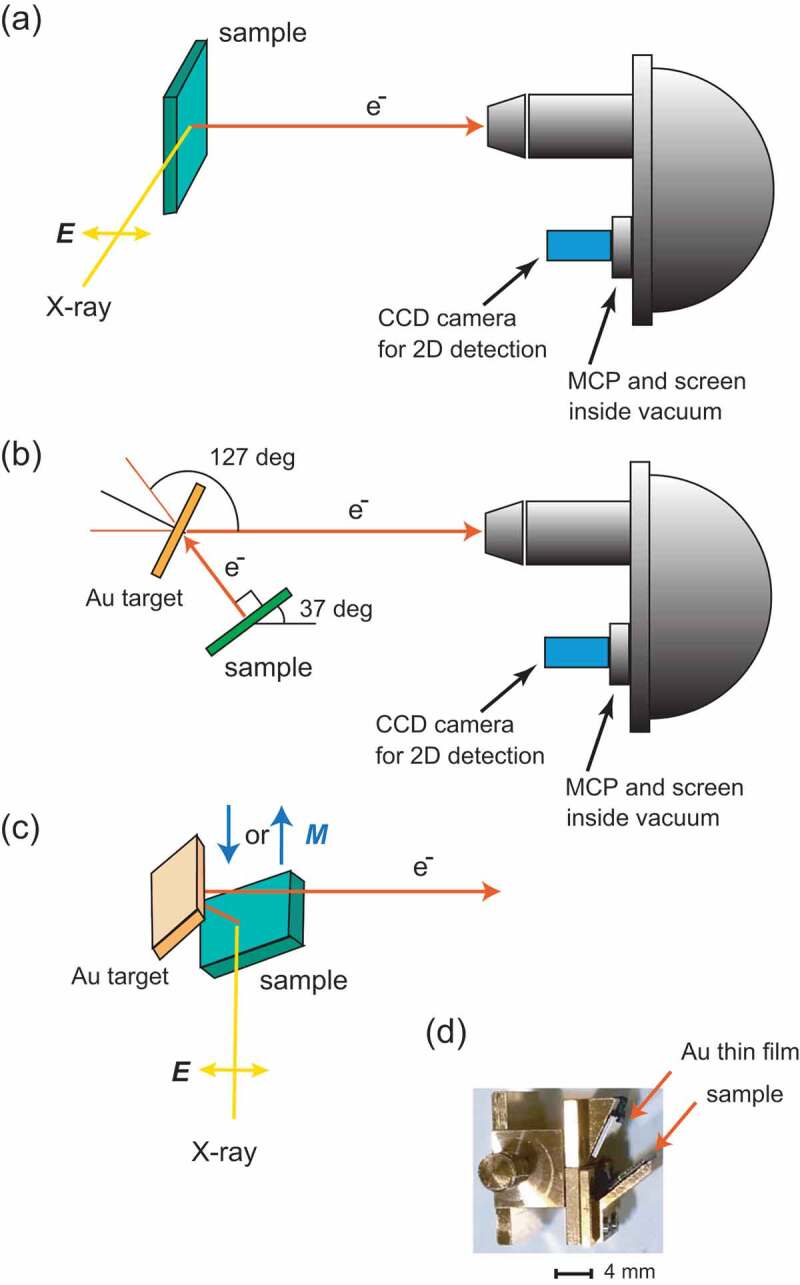
(a) Schematic illustration of the typical experimental geometry of HAXPES using a hemispherical analyser with a 2D multi-channel detector (combination of MCP, screen, and CCD camera). The E-vector (***E***) of X-ray is indicated by an arrow. (b) Schematic illustration of the experimental geometry of spin-resolved HAXPES in this work. The X-ray propagation direction is perpendicular to the illustration. Photoelectrons emitted from a sample scattered by a Au target are introduced into the hemispherical analyser. The incidence angle of photoelectrons to the Au target is set to 26.5°. The scattering angle of photoelectrons by the Au target is 127 ± 7°. (c) Schematic illustration of the sample and Au target viewing from top. The magnetisation direction (***M***) is indicated by arrows. (d) Photo of a sample carrier, which can install both thin films of sample and Au target simultaneously

Hard X-ray photoelectron spectroscopy (HAXPES) has been recognised as a powerful method for studying the bulk and interface electronic structures of materials owing to large inelastic mean-free–path of electrons (λ_e_) with the kinetic energy of several keV [1–4]. By utilising high-brilliance hard X-rays from undulators at third generation synchrotron facilities, the energy resolution (better than 0.3 eV in many cases) of HAXPES is comparable to that of soft X-ray PES, although the photoionization cross-sections of valence band and core-level electrons, e.g. the Fe 3*d* and 2*p*_3/2_ orbitals, respectively, for the hard X-ray region (~3-10 keV) are ~2-4 orders of magnitude lower than those for the soft X-ray (e.g. 1 keV) excitation [5,6] as seen in Figure 1. To enhance the photoemission intensity in HAXPES, the normal emission geometry with grazing incidence of well-focused X–rays from an undulator light source has been employed in many cases [1–4] as shown in Figure 2(a). Since the attenuation length of X-rays (λ_p_) with the energy of several-keV in solids is much larger than λ_e_ for several-keV photoelectrons, the grazing incidence of X-rays enhances the probability of excitation of electrons within the depth of λ_e_ from the surface, that is, λ_p_ approaches to λ_e_ with decreasing the incidence angle for the non-total reflection condition [7]. The use of the two-dimensional (2D) multi-channel detector which combines a multi-channel plate, screen, and CCD camera in a recent electron analyser (see Figure 2(a)) also contributes to high-efficient HAXPES experiments. In the 2D electron detection, one axis corresponds to the kinetic energy of electrons, and the other axis corresponds to sample position or detector angle, depending on detection modes.

Various methods used in vacuum ultraviolet (VUV) and soft X-ray PES are also realised in HAXPES experiments. For example, magnetic circular and linear dichroism (MCD and MLD) in HAXPES [8–11], X-ray standing-wave (XSW) HAXPES [12], hard X-ray angle-resolved PES (HARPES) for band dispersion [13,14], XSW-HARPES [15], HAXPES combined with X-ray total reflection [7], and spin-resolved HAXPES [10,11,16,17] have been reported in the last decade. Among them, spin-resolved HAXPES can be a potential candidate for the direct probe of the bulk-sensitive spin-dependent valence band electronic structures of magnetic materials. However, due to extremely low cross-section of the valence electrons in the hard X-ray region relative to the VUV and soft X-ray regions (see Figure 1), the reported spin-resolved HAXPES (spin-HAXPES) experiments have limited in the Fe 2*p* core–level of Fe based ferromagnetic materials [10,11,16,17]. In addition, the extremely low efficiency of a typical spin detector, such as a Mott detector [18–20] whose figure of merit (FOM) is in the order of 10^−4^, prevents us from accessing the spin-HAXPES experiments in the valence band region. While Kozina et al. [17] reported spin-HAXPES experiments in the vicinity of the Fermi-level (E_F_) for body-centred cubic FeCo(001), bulk-sensitive spin-HAXPES for the entire valence band region has not been reported until now. To realise the spin-HAXPES experiments for the valence band region, either the development of the X-ray light source or the improvement of FOM in the spin detector is required, since the extremely low cross-section and FOM represent the photon-hungry and electron-hungry experimental aspects, respectively.

Recently, in spin- and angle-resolved photoelectron spectroscopy (SARPES) using VUV light, high-efficiency (high FOM) spin detectors have been used for high-throughput and high-resolution experiments instead of the standard Mott detector. The very low energy electron diffraction (VLEED) type detector using the Fe(001)-*p*(1×-1)-O film [21] and the W(110) and Au/Ir(100) spin-filter [22,23] with the 2D multi-channel detection have been used in the SARPES experiments to enhance the efficiency of SARPES experiments. These works strongly suggest that the use of multi-channel spin detection is key for high-throughput SARPES experiments. Thus, we considered to use a multi-channel spin-filter in spin-HAXPES for high-throughput experiments as schematically shown in Figure 2(b,c). High-kinetic energy photoelectron with 6 keV emitted from the sample are scattered by a Au target film, and then the scattered electrons are analysed by a hemispherical electron analyser. This experimental setup allows us to perform the 2D multi-channel detection in spin-HAXPES. This scattering process involves the Mott scattering, and a Au film acts as a spin-filter in this case. In addition, we do not need to modify the electron analyser as seen in Figure 2(a,b), but need only to introduce a customised sample carrier as shown in Figure 2(d), which can mount thin films of sample and Au simultaneously.

## 2. Experiment

(001)-oriented epitaxial Fe thin films with 50 nm thickness were grown on MgO(001) substrates using an ultrahigh vacuum (UHV) magnetron sputtering system. The base pressure in the deposition chamber was better than 1 × 10^−7^ Pa. First, the surfaces of MgO substrates were cleaned by *in situ* Ar ion sputtering, followed by a flushing process at 600 °C for 30 minutes. Then, Fe(50 nm) layer was deposited at room temperature (RT) and annealed at 300 °C in UHV to improve crystallinity and surface flatness of the Fe layer. Finally, a 2-nm-thick MgO layer was formed on the Fe films at RT to protect the Fe films from oxidation in air. The (001)-oriented epitaxial growth and low surface roughness (average roughness was 0.15 nm) were confirmed by X-ray diffractometer and atomic force microscopy, respectively. The magnetisation curve measured to <110> and <100> in-plane directions of the Fe films revealed that almost perfect remanence magnetisation appeared in the <100> (<110>) direction of the Fe films (MgO substrates). Epitaxial 50-nm-thick Au(001) films were also fabricated by UHV magnetron sputtering system on Cr(5 nm)-buffer/MgO(001) substrate structure.

The MCD-HAXPES and spin-HAXPES measurements were conducted at the undulator beamline BL15XU [10,24] of SPring-8. The photon energy was set at 5.95 keV. Total energy resolution for MCD-HAXPES was set to 0.24 eV, while that for spin-HAXPES was set to 0.68 eV at RT. The binding energy (E_B_) was calibrated by E_F_ of an evaporated Au film. To remanently magnetise the Fe film along the <100> direction, the magnetic field of ~0.3 T was *in situ* applied by a Nd-Fe-B based permanent magnet in an analysis chamber of the HAXPES apparatus. To analyse and detect the photoelectrons, a hemispherical electron analyser (VG Scienta R4000) was used in an angle-integrated transmission mode with the acceptance angle of ± 7°. For the MCD-HAXPES measurements, horizontal linear polarised (H-pol.) X-rays were converted to left- or right-handed circularly polarised (LCP or RCP) X-rays by using a diamond phase retarder. The experimental geometry for MCD-HAXPES was the same as that shown in Figure 2(a), except for the X-ray polarisation. The incidence angle of X-rays was set to about 2° with respect to the sample surface. For the spin-HAXPES measurements, the experimental geometry shown in Figure 2(b,c) with H-pol. X-rays was employed. The magnetisation direction of the samples was periodically reversed during the measurements to obtain the spin–resolved valence band spectra, while the standard Mott detector, which has in general four detection channels sensitive to the left, right, up, and down spin components, does not require the magnetisation reversal of the samples if the detector has been optimised to reduce the extrinsic intensity asymmetry between the detection channels [18–20]. In our proposed spin-HAXPES, in principle, we have to measure two spectra with the sample magnetisation nearly parallel and antiparallel to the X-ray propagation direction as shown in Figure 2(c) to obtain the spin resolution. The incidence angle of H-pol. X-rays was set to about 5° with respect to the sample surface and magnetisation direction. The photoelectrons scattered by the Au thin film with the scattering angle of 127 ± 7° as shown in Figure 2(b) were analysed and detected by the electron analyser. The normal emission geometry was employed in the HAXPES experiments (see Figure 2(a,b)).

## 3. Results and discussion

**Figure 3.**
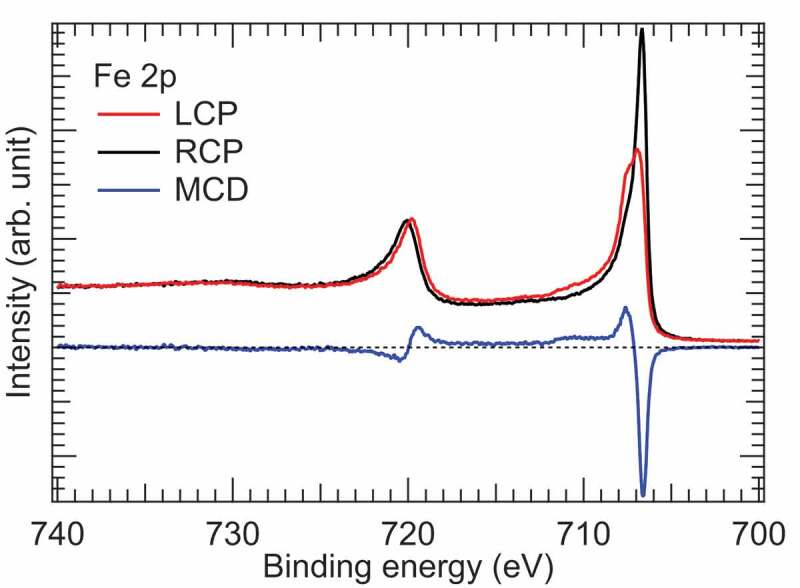
Fe 2*p* core-level HAXPES spectra of the buried Fe thin film measured with LCP and RCP. MCD spectrum is the intensity difference between the HAXPES spectra for LCP and RCP

Before conducting the spin-HAXPES measurements, we have measured the Fe 2*p* core-level MCD-HAXPES to confirm the magnetisation of the Fe film in the experimental geometry shown in Figure 2(a). Figure 3 shows the Fe 2*p* core-level HAXPES spectra measured with LCP and RCP X-rays. The intensity difference between the spectra corresponds to MCD. The observed Fe 2*p* core-level HAXPES and MCD spectra are similar to those reported in the previous work [9]. One sees huge MCD signals in the Fe 2*p*_3/2_ region, indicating that the remanent magnetisation is sufficiently large in the Fe film after applying a magnetic field of ~0.3 T.

Next, we assessed the effective Sherman function (*S_eff_*) and FOM in spin-HAXPES with the geometry shown in Figure 2(b,c). To evaluate the scattering probability (*I*/*I*_0_) of the Au target, the Au thin films were placed at both the target and sample positions. Then, the intensity of Au 4 *f* photoelectrons emitted from the Au target was measured when the target was irradiated by X-rays. The Au 4 *f* photoelectrons, which were emitted from the Au thin film irradiated by X-rays, scattered by the target were also measured. By considering the experimental geometry, X-ray polarization, X-ray intensity, and field of view of the electron analyser at the Au target, the *I*/*I*_0_ was evaluated to be ~0.04, where *I*_0_ is the photoelectron intensity in the field of view of the electron analyser at the Au target, and *I* is the scattered photoelectron intensity by the Au target for *I*_0_. We note that the field of view of the electron analyser at the Au target strongly restricts the photoelectron intensity in the experimental geometry shown in Figure 2(b,c). For the standard experimental geometry (see Figure 2(a)), the photoelectron intensity detected by the analyser is correlated with the field of view of the electron analyser and the footprint of X-rays on the sample surface, that is, an X-ray footprint on the sample surface which is larger than the field of view restricts the detectable photoelectrons emitted from the sample for the standard experimental geometry. For the geometry shown in Figure 2(b,c), part of the photoelectrons emitted from the sample can reach the field of view at the Au target; in this situation, the photoelectron intensity reduces to 2% in comparison with that for the standard experimental geometry (*I*_std_), leading *I*_0_ = 0.02 × *I*_std_.

**Figure 4.**
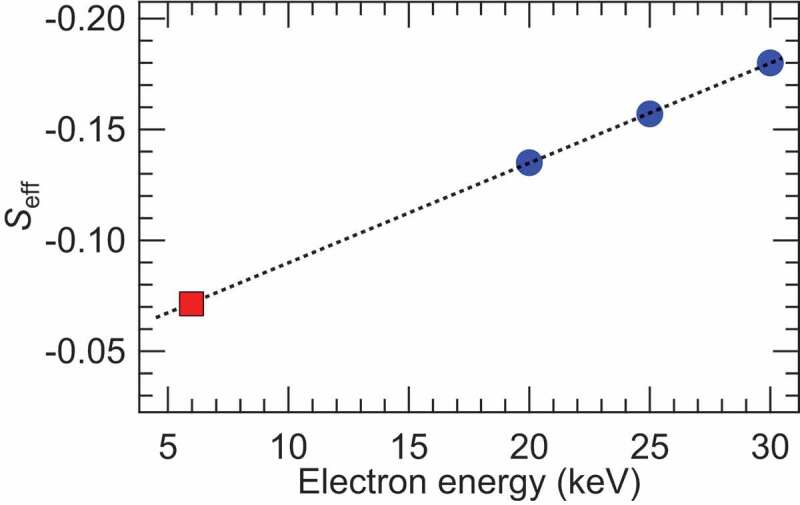
*S_eff_* as a function of the electron energy. The blue circles indicate the experimental *S_eff_* of the medium energy Mott detector taken from Ref [19]. The black dotted line is obtained by the least square fitting for the blue circles. The red square is plotted on the black dotted line at the electron energy of 5.95 keV

The *S_eff_* of −0.07 for 5.95 keV electrons was obtained from the linear extrapolation of the *S_eff_* in the medium energy Mott-type spin-detector for 20, 25, and 30 keV [19] for simplicity as shown in Figure 4, since the Sherman function was almost linear as a function of the electron energy below 30 keV for the Mott scattering [18]. This low *S_eff_* is due to the plural and multiple scattering, which reduces the value of *S_eff_*, in Au for lower electron energy below 30 keV [18,19]. The single-channel FOM given by *S_eff_*^2^(*I*/*I*_0_) was evaluated to be ~2 × 10^−4^. This value is comparable to the typical Mott–type spin detectors [18–20]. Since we can utilize the 2D multi-channel detector in spin-HAXPES as used in the standard HAXPES measurements, the effective FOM is enhanced by a factor of ~4 × 10^4^, which is the number of active channels of the electron detector (427 channels in the position axis × 92 channels in the energy axis in the 2D detector) in the spin-HAXPES measurements, in comparison with the case when only 1 channel of the 2D detector is used.

**Figure 5.**
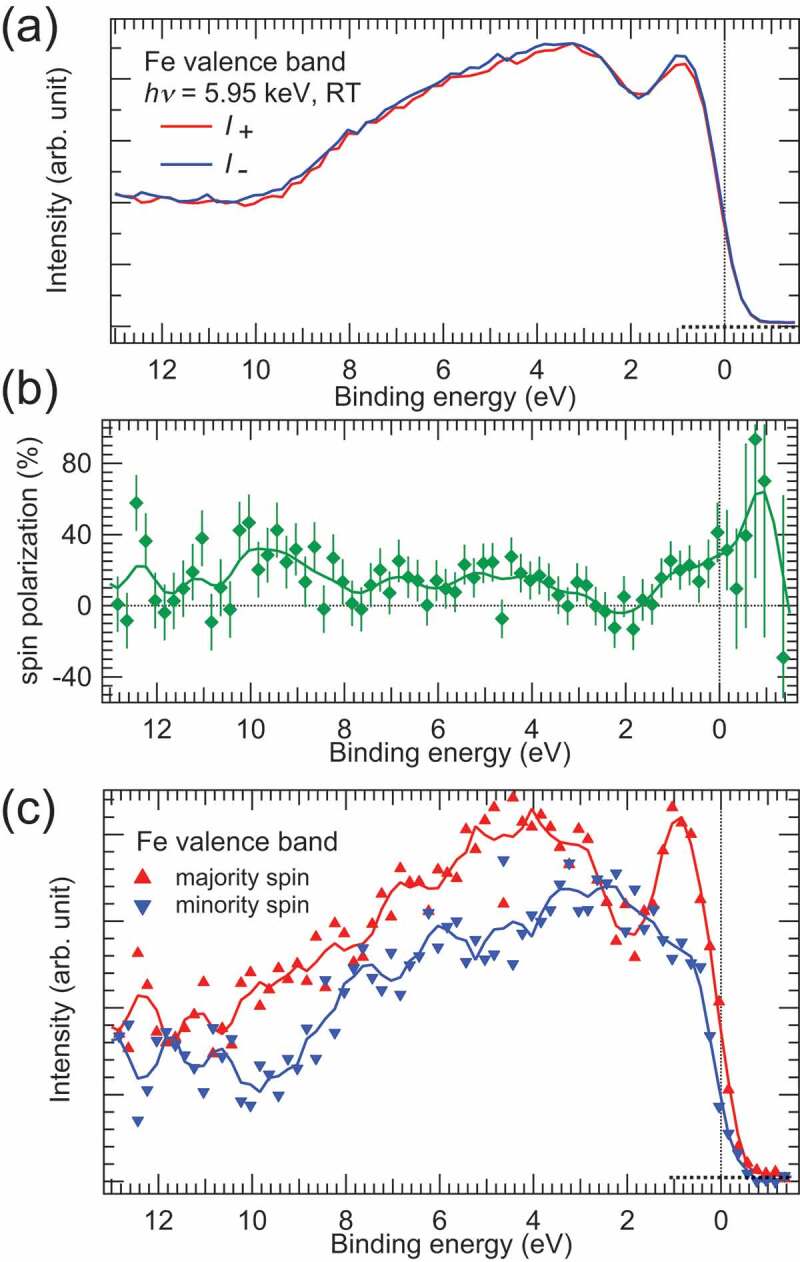
(a) Valence band HAXPES spectra (*I*_+_ and *I*-) of the buried Fe thin film. Here, the spectra of *I*_+_ and *I*- were measured with the remanent magnetisation, being antiparallel and parallel to the X-ray propagation direction. (b) Spin polarisation spectrum of the Fe film in the valence band region. Vertical bars represent statistical errors. The unit is given in percent. The solid curve is to guide the eye. (c) Spin-resolved valence band HAXPES spectra of the Fe thin film. The solid curves are to guide the eye. Total data acquisition time is ~13 hours

Figure 5(a) shows the valence band HAXPES spectra of the Fe film measured with the magnetisation nearly antiparallel (*I*_+_) and parallel (*I*-) to the X-ray propagation direction in the experimental geometry shown in Figure 2(b,c). The H-pol. X-rays are used to excite the photoelectrons from the valence band region. The spectra of *I*_+_ and *I*- are similar each other, while the spectral shapes are slightly different. The peak structure at E_B_ of ~1 eV mainly arises from the Fe 3*d* states, while the broad peak structure in the region between 2 and 8 eV mainly arises from the Fe 4*s* states according to Ref [25]. The strong Fe 4*s* photoemission intensity originates from the higher photoionization cross-section of the Fe 4*s* orbital than the Fe 3*d* orbital for the photon energy of 6 keV [6,26,27]. One may think that these spectra involve the energy loss component due to the excitation of plasmons in the Au target. We examined the energy loss probability and loss energy due to the plasmon from the Au 4 *f* core-level spectrum measured with 5.95 keV X–rays (not shown), and found that the plasmon energy is 6.3 eV and the loss probability is 0.75%. Thus, we can conclude that the contribution of the energy loss component due to the plasmon in the Au target to the valence band spectra of *I*_+_ and *I*- is negligibly weak.

We calculated the spin polarisation (*P*) spectrum shown in Figure 5(b) using the *I*_+_ and *I*- spectra as follows,

(1) $$P=\;\left(I_{+}-I_{-}\right)\;/\;\left(I_{+}+I_{-}\right)\;/S_{eff},$$

with *S_eff_* = −0.07. The spin polarisation spectrum showed that large spin polarisation near E_F_ and at E_B_ of ~ 5 eV, which mainly arose from the Fe 3*d* and 4*s* states, respectively, by taking account of the spin-resolved valence band structure of bulk Fe (see Ref [25].). Then, the spin-resolved HAXPES spectra, which correspond to the majority (*I_maj_*) and minority (*I_min_*) spin-resolved valence band spectra, were obtained by using the *P, I*_+_ and *I*- spectra as follows,

(2) $$I_{maj}=\;\left(I_{+}+I_{-}\right)\times \left(1\;+P\right)/2,$$

(3) $$I_{min}=\;\left(I_{+}+I_{-}\right)\times \left(1\;-P\right)/2.$$

Figure 4(c) shows the spin-resolved valence band HAXPES spectra of the buried Fe film. The sharp peak at E_B_ of ~1 eV and the broad structure at E_B_ of ~4 eV were found in the majority spin states, while fine structures were not found in the minority spin states. The background intensity due to the secondary electrons in the high E_B_ side is different between the majority and minority spin spectra, since the spin polarisation takes place in the case of the secondary electrons. As mentioned above, the Fe 3*d* states are mainly located near E_F_ and the broad Fe 4*s* states are located in the region between 2 and 8 eV. Therefore, we see that the sharp peak located at E_B_ of ~1 eV arises from the Fe 3*d* majority spin states, and the broad structure at E_B_ of ~5 eV arises from the Fe 4*s* majority spin states. The Fe 3*d* and 4*s* minority spin states shift to the lower E_B_ side by ~2 eV relative to the majority spin states due to the exchange splitting in Fe. Therefore, a sharp peak for the Fe 3*d* minority spin states is placed in the energy above E_F_ (see Ref [25].), and is not seen in the spin-HAXPES spectra.

**Figure 6.**
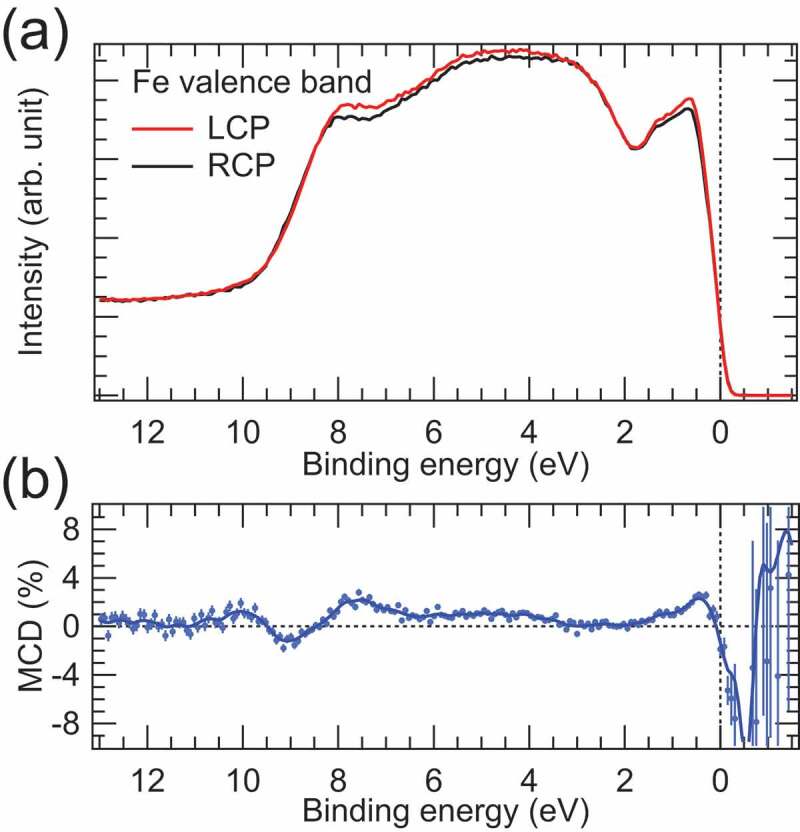
(a) Valence band HAXPES spectra of the buried Fe thin film measured with LCP and RCP. (b) MCD spectrum obtained from the intensity asymmetry of the HAXPES spectra for LCP and RCP. The unit is given in percent. The solid curve is to guide the eye. Vertical bars represent statistical errors

One could suspect that the spin polarisation and related spin-resolved spectra are originated from a kind of magnetic dichroism in HAXPES. To exclude this possibility, we have performed the MCD-HAXPES measurements in the valence band region. Figure 6(a) shows the valence band HAXPES spectra of the Fe thin film measured with LCP and RCP X-rays. Since the energy resolution is better in MCD-HAXPES than spin-HAXPES, the valence band spectra shown in Figure 6(a) is sharper than those shown in Figure 5(a). The shoulder structure at E_B_ of ~8 eV is due to the O 2*p* states of the top MgO layer. The intensity asymmetry of the valence band spectra measured with LCP and RCP X-rays corresponds to MCD as shown in Figure 6(b). One sees that the MCD signal is very weak in the entire valence band region and the MCD profile is different from the spin polarisation profile shown in Figure 5(b). The weak MCD signal is due to the weak spin-orbit interaction of the valence electrons in Fe, because the origin of MCD in PES is the combination of the spin-orbit interaction and dipole transition selection rule for the circularly polarized light. Thus, we can conclude that the spin polarisation spectrum shown in Figure 5(b) reflects the genuine spin-dependent electronic states of Fe.

To further validate the observed spin polarisation and spin-resolved HAXPES spectra of Fe, we have calculated the spin-resolved cross-section weighted density of states (CSW-DOS) of Fe using the photoionization cross-sections and partial DOSs for the Fe 3*d*, 4*s*, 4*p* states. The cross-sections were calculated for the experimental geometry shown in Figure 2(b) according to Refs. [6,26,27], and partial DOSs were obtained from Ref [25]. The cross-section ratios of the 4*s* and 4*p* to 3*d* orbital were set to 29.67 and 0.92, respectively, for the experimental geometry in spin-HAXPES. Here, the calculated 4*s* and 4*p* cross-sections were multiplied by factors of 2.5 and 3.0, respectively, to better reproduce the experimental spectra. The broadening procedure was applied to the spin-resolved CSW-DOSs for taking the energy resolution and lifetime broadening into account (see e.g. Refs. [9,25]). The calculated spin-resolved HAXPES spectra were obtained from the sum of the 3*d*, 4*s*, and 4*p* spin-resolved CSW-DOS spectra for simplicity. Then, the spin polarisation spectrum was deduced from the calculated spin-resolved HAXPES spectra.

**Figure 7.**
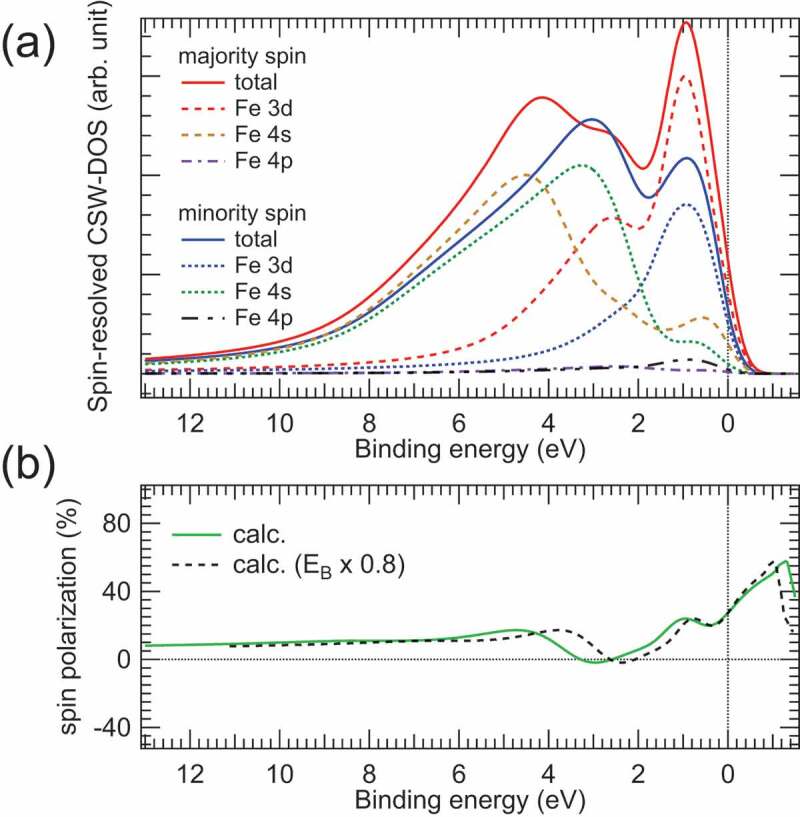
(a) Calculated spin-resolved CSW-DOSs of Fe in the valence band region. The spin-resolved Fe 3*d*, 4*s* and 4*p* CSW-DOSs are also shown in the figure. (b) Calculated spin polarization (solid line) deduced from the spin-resolved CSW-DOSs shown in (a). The dashed line in (b) represents that the energy scale of the calculated spin polarization is multiplied by a factor of 0.8

Figure 7 shows the calculated spin-resolved HAXPES and spin polarisation spectra of Fe. For comparison, the spin-resolved 3*d*, 4*s*, 4*p* CSW-DOS spectra are also shown in the figure. At a glance, the 3*d* and 4*s* components dominate the calculated spin-resolved HAXPES spectra. Again, the large 4*s* orbital contribution to the HAXPES spectra is due to the relatively large 4*s* cross-section in the hard X-ray region [6,26,27], although the 4*s* partial DOS itself is very small in Fe. The 4*p* orbital contribution is very weak due to both small 4*p* partial DOS and small 4*p* cross-section [25]. One sees that the calculated spin-HAXPES spectral shapes are in good agreement with the experimental ones shown in Figure 5(c). The calculated spin polarisation and spectral shape are also consistent with the experimental ones shown in Figure 5(b). Although the statistical error is large in the present experimental results, we can conclude that the valence band spin-HAXPES spectra and spin polarisation from the buried Fe film are successfully observed by our proposed method.

We see that the calculated spin-resolved HAXPES spectra are very similar to the experimental ones in the case of ΔE = 0.68 eV. In contrast, the high-resolution spin- and angle-integrated valence band HAXPES spectra for the thick Fe film and bulk Fe deviate from the calculated HAXPES (CSW-DOS) spectrum in the case of ΔE = 0.24 eV as can be seen in Refs. [9,25]. Particularly, there is obvious deviation in the spectral shapes originating from the Fe 3*d* states near E_F_ between the calculated and experimental spectra. Since the spin-HAXPES experiments are conducted with the low energy resolution (0.68 eV), the deviation seen in the high-resolution HAXPES and calculated spectra (0.24 eV) might be rounded. However, it can be seen that E_B_’s of the shoulder (~1 eV) and valley (~2 eV) structures between the experimental and calculated spin polarisation spectra are slightly different (see Figures 5(b) and 7(b)). Even though the energy resolution is low in the present spin-HAXPES measurements, the spin resolution allows us to detect the deviation between the experiments and calculations. When the energy scale of E_B_ for the calculated spin polarisation spectrum is simply reduced by 20% (indicated by black dashed lines in Figure 5(b)), the deviation between the experimental and calculated results is reduced. This result suggests possible bandwidth narrowing due to the electron correlation effects, as has been reported in the theoretical calculations [28,29] compared with the surface-sensitive angle-resolved photoelectron spectroscopy data for Fe. Thus, we see that the electron correlation effects are of importance for the spin-resolved electronic states of Fe even in the bulk region probed by bulk-sensitive spin-HAXPES. To clarify the electron correlation effects on the spin-resolved band structures of bulk Fe measured by spin-HAXPES in detail, theoretical analysis with the combination of the dynamical mean field theory and one–step model of photoemission [30–32] is required.

In the case of HAXPES measurements using several keV X-rays at RT, even ultrahigh-angular resolution experiments, e.g. better than 0.01°, cannot detect the band dispersion of single crystals. This is because the photoemission Debye-Waller (DW) factor, which is a fraction of the momentum-conserved transition (so–called direct transition) [13,33–35], is extremely low for HAXPES at RT. The DW factor for Fe is calculated to be ~10^−12^ in the case of the photon energy of 6 keV at RT. Even at 30 K, the DW factor for Fe is calculated to be ~0.06. The low DW factor means that the valence band spectra shown in Figure 5 are governed by non-direct transitions for Brillouin-zone (BZ) averaging. In contrast, HARPES with the lower excitation X-ray energy (~3 keV) and lower temperature (~30 K) allows us to observe the band dispersion for many materials, since the DW factor increases with decreasing both the excitation X-ray energy and sample temperature as has been reported in Refs. [4,13,14].

It is known that recoil effects on the photoelectrons occur in HAXPES with several keV X–rays, in particular for light elements [36–38]. The calculated recoil energy of photoelectrons with the kinetic energy of 6 keV for Fe is 58.4 meV [25], which is smaller than the experimental ΔE of MCD- and spin-HAXPES. Although the recoil effects cause shifting and broadening of the HAXPES spectra, it is difficult to detect such effects within our experimental resolution. However, the recoil effects would also contribute to the BZ averaging through the energy recoil processes in photoemission. The BZ averaging effect in HAXPES seems to make spin- and angle-resolved HAXPES unsuitable for observing the bulk-sensitive band dispersion for magnetically ordered single crystalline materials. But the BZ averaging effect in HAXPES is expected to be suitable for the direct determination of half-metallicity for the potential half-metals in single crystal form, since the exploring of half-metallicity by SARPES with VUV or SX light source with the fixed photon energy is governed by direct transitions and the observable momentum space is limited by the photon energy, particularly the momentum-conserved transition along the surface normal direction of materials. Therefore VUV- and SX-SARPES may miss observing suitable or unsuitable momentum space on spin polarisation. Owing to the bulk-sensitivity and BZ averaging in spin-HAXPES, we were able to observe the genuine spin-dependent electronic structures and spin polarisation from the buried Fe film.

As can be seen in Figure 5, the statistical error is large in the present spin-HAXPES measurements, while we have used the hemispherical analyser with the 2D multi-channel detector to enhance the detection efficiency. The use of 2D multi-channel detection increases the effective FOM, but the mismatch of the analyser field of view at the Au target position in our experiments reduces the number of photoelectrons entering the analyser as mentioned above. By maximizing the photoelectrons entering the analyser by tuning the lens parameters to widen the analyser field view, the statistical error and energy resolution of spin-HAXPES will be improved.

## 4. Summary

We have developed spin-HAXPES using the ultracompact Mott-type spin-filter, which allows us to use the benefits of the 2D multi-channel detection of the electron analyser without modifying the HAXPES apparatus. The *S_eff_* and single-channel FOM were evaluated to be −0.07 and 2.0 × 10^−4^, respectively. Owing to the 2D detection, the effective FOM was enhanced by a factor of ~4 × 10^4^ compared to the case when only 1 channel of the 2D detector is used. The valence band spin-HAXPES spectra for the buried Fe film were demonstrated by our proposed method. The observed spin-HAXPES spectra and spin polarisation were fairly reproduced by the calculated spectra obtained from the sum of the cross-section weighted Fe 3*d*, 4*s*, and 4*p* partial DOSs. We confirmed that observed spin-HAXPES spectra are not caused by a kind of magnetic dichroism by comparing the spin polarisation and MCD spectra. In the spin polarisation spectra, the small deviation in the energy scale was found between the experiment and calculation. This deviation suggests that the electron correlation effects are key for the spin-resolved electronic states of bulk Fe. Thus, spin-HAXPES is a useful probe for detecting genuine spin-resolved valence band electronic states of buried magnetic materials. Further development of the efficiency in spin-HAXPES is required to obtain the high-statistical and high-resolution experimental spin-resolved electronic structures of various functional magnetic materials.
